# Nanomechanical Atomic Force Microscopy to Probe Cellular Microplastics Uptake and Distribution

**DOI:** 10.3390/ijms23020806

**Published:** 2022-01-12

**Authors:** Farida Akhatova, Ilnur Ishmukhametov, Gölnur Fakhrullina, Rawil Fakhrullin

**Affiliations:** Institute of Fundamental Medicine and Biology, Kazan Federal University, Kreml uramı 18, Kazan 420008, Republic of Tatarstan, Russian Federation; akhatovaf@gmail.com (F.A.); irishmukhametov@gmail.com (I.I.)

**Keywords:** atomic force microscopy (AFM), human skin fibroblasts (HSF), microplastics, nanoplastics, dark-field hyperspectral microscopy, nanomechanical characteristics

## Abstract

The concerns regarding microplastics and nanoplastics pollution stimulate studies on the uptake and biodistribution of these emerging pollutants in vitro. Atomic force microscopy in nanomechanical PeakForce Tapping mode was used here to visualise the uptake and distribution of polystyrene spherical microplastics in human skin fibroblast. Particles down to 500 nm were imaged in whole fixed cells, the nanomechanical characterization allowed for differentiation between internalized and surface attached plastics. This study opens new avenues in microplastics toxicity research.

## 1. Introduction

Microplastics pollution is currently regarded to be among severe environmental threats [1], it is clear by now that most, if not all, habitats are polluted with microplastic to a certain extent [2]. The mechanisms of microplastics uptake by organisms and subsequent toxicity are not fully understood yet [3], therefore it is challenging to investigate the effects of microplastics in vitro, using uniform and chemically consistent particles as model microplastics. Polystyrene microbeads have become an important tool in microplastics toxicity studies due to their controllable shape, narrow size distribution and commercial availability [4]. Although it is clear that polystyrene microbeads are not fully representative as other types of microplastics [5], especially the environmental specimens, which may and do have a range of sizes, shapes and chemical composition, in the first approximation the polystyrene microbeads have been employed in a number of the microplastics-related studies, especially aimed at elucidation of biological activity in vitro. In particular, polystyrene microbeads have been employed in experiments to elucidate the microplastics toxicity using several human cell cultures [6,7,8,9] and multicellular organoids [10]. Moreover, in vivo studies also rely on spherical polystyrene microparticles, as demonstrated in a number of recent reports [4,11].

Developing effective methods to visualise and identify micro- and nanoplastics is of paramount importance in modern bioanalytical chemistry [12], since understanding of pollution extent in natural habitats and toxicity pathways in cells and organisms is impossible without quantitative identification of polymeric solids [13], which are exceptionally diverse and hard to isolate from complex biological matrices. Spectroscopy techniques, such as FTIR or Raman microspectroscopy [14] are routinely used to identify microscale plastics in environmental samples, which are rarely used to investigate mechanisms of microplastics toxicity due to their complex chemistry, shape and size. Microscopy observations are particularly important for evaluation of commercially-available submicron plastics (e.g., polystyrene nano- and microparticles) cellular uptake and biodistribution [15], which requires fluorescence labelling of polystyrene prior to ingestion by cells followed by fluorescence wide-field or confocal microscopy [16,17]. Pre-treatment or post-treatment with polymer-targeting fluorescent dyes is essential for visualisation of cellular uptake and intracellular distribution of commercial spherical polystyrene microbeads [18], whereas label-free techniques utilising infrared [19,20] or Raman [21] microspectroscopy are mostly reserved for diverse environmental samples, where the chemical composition of the particles analysed is unknown. Recently, label-free detection of polystyrene microplastics by means of reflected light hyperspectral microimaging [22] and dark-field microscopy along with neural networks [23] were developed for in vitro and in vivo applications.

Atomic force microscopy, as a primarily surface characterisation technique employing the interaction of a thin probe with a surface [24], has recently found some limited applications in microplastics research. First, nanoscale topography imaging and quantitative roughness analysis, intrinsic for AFM, was used to investigate the surfaces of various microplastics [9]. For example, roughness of polymer planar surfaces immersed into seawater was studied [25], indicating the microplastics formation. Surface topography during the formation of microbial biofilms on surfaces of environmental short chain low-density polyethylene was investigated [26]. Next, AFM in combination with infrared spectroscopy was utilised to visualise and chemically identify microplastics isolated from tap water [27]; evaluate the microbial biofilms induced plastic surfaces deterioration [28]; asses the thermal effects on microplastics surface properties [29] and photoageing of microplastics [30]. However, to the best of our knowledge, there were no reports on imaging of microplastics in human cells using AFM. This is not surprising, taken the fact that AFM is not fully suitable to probe the internal parts of biological entities, whereas the detection of microplastics exclusively on cell membranes is of lesser importance. Nevertheless, we hypothesized that using AFM in nanomechanical mode (that is where the image acquisition is followed by quantitative measurements of such parameters as modulus, adhesion, etc.) might be applicable for detection of microplastics taken up by human cells if the latter are chemically fixed and dried, following the routine AFM sample preparation protocol. In this case, the solid spherical microplastics will be an easy target for the tip of AFM probe, having spatial geometry and mechanical properties different from the adjacent cellular compartments. In this Communication we demonstrate the feasibility of our technique, using human skin fibroblasts primary cells, submicron polystyrene microbeads and PeakForce Tapping nanomechanical AFM.

## 2. Results

### 2.1. Polystyrene Microplastics Cytotoxicity Evaluation

**Figure 1:**
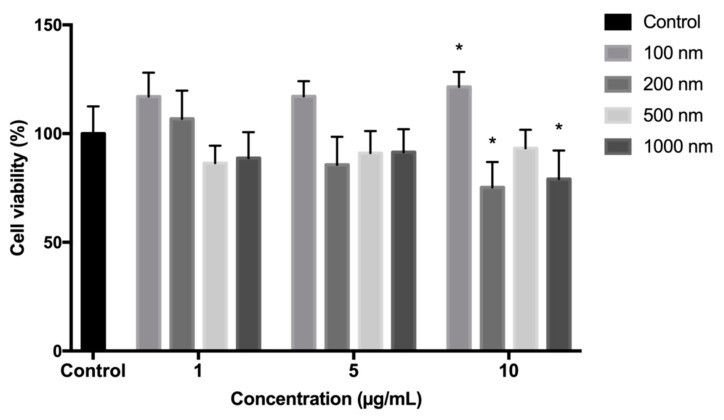
The effects of polystyrene particles (at increasing diameters) on cell viability measured by MTT assay. HSF were exposed to 1, 5 and 10 µg/mL of particles for 24 h. * *p* < 0.05 related to the untreated control group, using one-way ANOVA followed by Dunnett’s test.

First, we evaluated the toxic effects of polystyrene microbeads on human skin fibroblasts (HSF), since no previous study, to the best of our knowledge, investigated this combination of cells/microplastics, although HSF were used earlier to evaluate polyethylene microbeads cytotoxicity [31]. The influence of PS particles at a different concentration on HSF viability was assessed using an MTT assay. As shown in Figure 1, no significant cytotoxicity (*p* > 0.05) was observed following exposure to all polystyrene microparticles at 1 and 5 µg/mL for 24 h. At 10 µg/mL, 200 and 1000 nm particles reduced the viability of cells to 75.2 ± 11.7% and 79.1 ± 13.1%, respectively. Conversely, 100 nm particles even stimulated cell viability to 121.5 ± 6.9% at 10 µg/mL. These results correlate well with a recent study where mouse embryonic fibroblasts were subjected to 100 nm polystyrene beads, which also did not affect the viability and proliferation of the cells [32]. However, other polystyrene beads of other sizes were not investigated using fibroblasts cell cultures, therefore we indicate here that the size-dependent effects are likely and need to be studied in more detail in the future.

In order to investigate cellular morphology after long-term incubation (24–72 h) with microplastic particles, we employed tetramethylrhodamine B isothiocyanate (TRITC) conjugated with Phalloidin and 4′,6-diamidino-2-phenylindole dihydrochloride (DAPI) for the visualisation of F-actin and nuclei of cells, respectively (Figure S1A–F). Additionally, we analysed cell morphology using AFM (Figure S1G–I). It is seen that HSF have a distinctive flat and spindle-shaped morphology with highly organised F-actin, indicating no visible cytoskeleton alterations after 24–72 h microplastic exposure at the 10 µg/mL concentration. At the same time, there are “micronucleus-like” structures in some cells of 24 and 72 h groups, which is probably to be the result of the nuclear budding process. Although the presence of these structures in small numbers is also observed in healthy cells [33], their formation may signal the possible genotoxic effect of particles. Thus, we postulate that further studies focused on assessing the genotoxicity of 500 nm polystyrene microparticles are required.

### 2.2. Microplastics Characterisation and Uptake Imaging with Dark-Field Hyperspectral Microscopy

**Figure 2:**
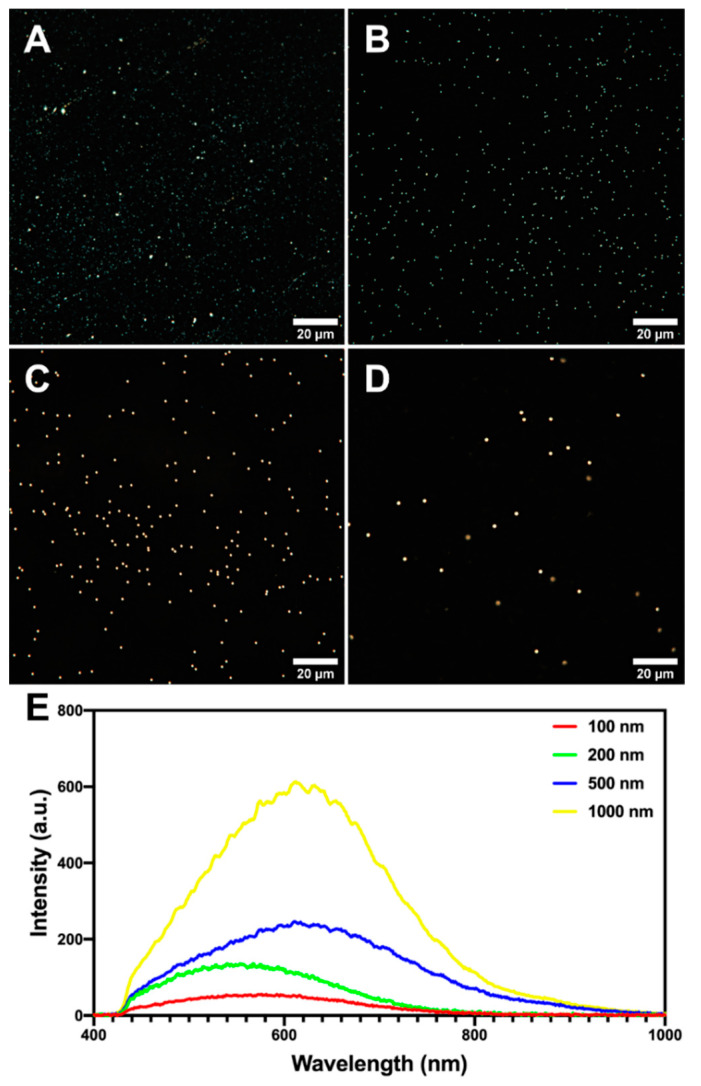
Dark-field images of (**A**) 100, (**B**) 200, (**C**) 500, (**D**) 1000 nm polystyrene particles in aqueous solution and (**E**) corresponding reflected/scattered Vis-near IR light spectra, obtained from hyperspectral images.

**Figure 3:**
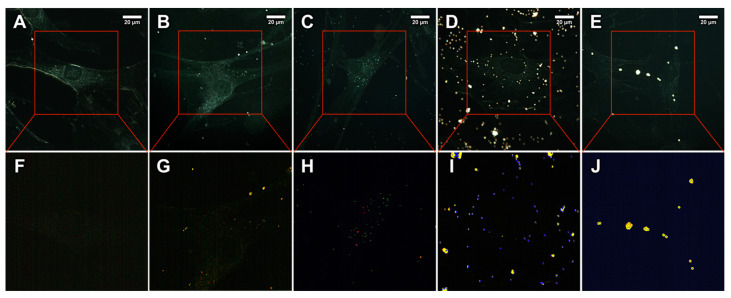
Dark-field images (upper row) and corresponding hyperspectral images merged with maps (bottom row) demonstrating the distribution of polystyrene particles in HSF after 24 h of co-incubation: control (**A**,**F**); 100 nm (**B**,**G**); 200 nm (**C**,**H**); 500 nm (**D**,**I**); and 1000 nm (**E**,**J**). Red-, green-, blue- and yellow-coloured pixels on hyperspectral images denotes 100, 200, 500, and 1000 nm particles mapped by Spectral Angle Mapper, respectively.

Here we employed our recently reported methodology to investigate nanoplastics using dark-field hyperspectral microscopy [22]. Dark-field images confirmed the uniform spherical morphology of 200, 500, and 1000 nm particles, while 100 nm particles appeared partly aggregated (Figure 2A–D). The reflected/scattered spectral libraries consisted of mean values of 10 particles for each sample were created (Figure 2E). It is seen that with the increase of the particle diameter, the scattering features also increased. Unlike in our previous study [22], herein we did not subtract the white halogen lamp spectrum, therefore the shape of spectral curves was different from previously reported, although in this case we also managed to identify the particles in cells using the spectral mapping due to characteristic features having an apparent etaloning-like features. Human skin fibroblasts were then exposed to 10 µg/mL of polystyrene particles, as seen in microscopy images the cells exhibited normal morphology (Figure 3A–E). Notably, as shown on the hyperspectral image of the control group (Figure 3F), the scattered light from cells is not that intensive as the light scattered from the plastic particles in the exposed cells. To confirm cellular uptake of 500 nm polystyrene particles, we captured the process of fine focus adjustment, revealed intracellular localisation of some particles (Videos S1 and S2). The further spectral matching application on hyperspectral data in the same field of view allowed to map image pixels attributed to the polystyrene particles (Figure 3F–J). The pixels similar to the spectrum of 100, 200, 500, or 1000 nm particles were colour-coded as red, green, blue, and yellow, respectively. Notably, in the control group no pixels were mapped indicating the absence of any polystyrene. The particles in treated cells were correctly classified as particles of the appropriate diameter used for the particular cell sample. However, some particles were expectedly misclassified in all hyperspectral images due to the identical structure of particles and quite similar spectral profiles. Thus, in the 100, 200, and 500 nm groups, the most intense particles were partly classified as 1000 nm spectra. Pixels in the centre of 1000 nm particles were classified as 500 nm. Generally, the dark-field microscopy coupled with the spectral matching technique allows to identify of particles, differing only in diameter, in the cells [23].

### 2.3. Microplastics Characterisation and Uptake Imaging with Atomic Force Microscopy

**Figure 4:**
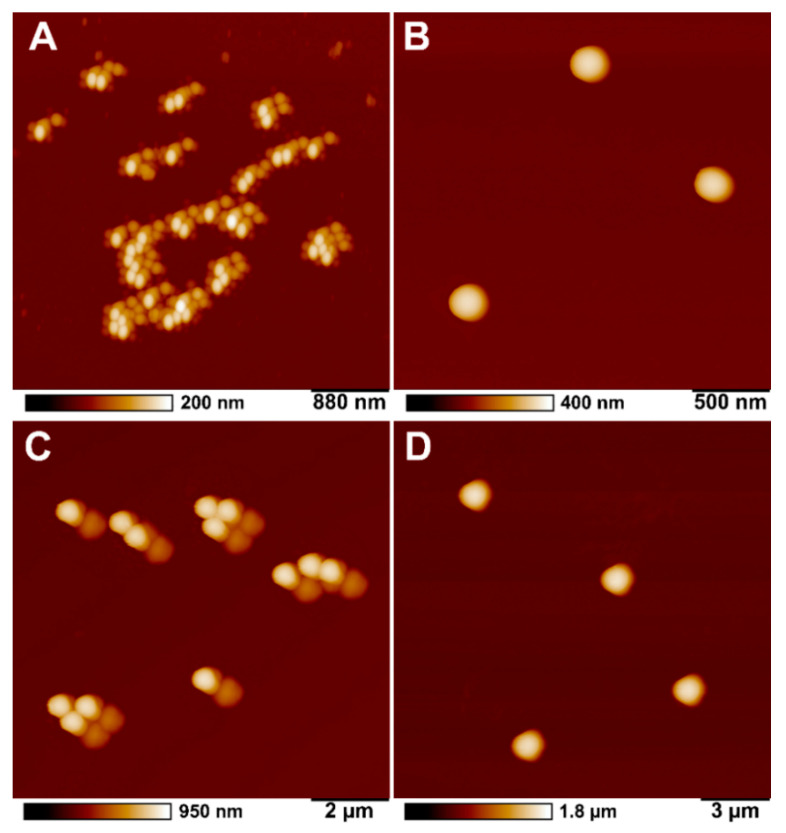
AFM images of (**A**) 100 nm, (**B**) 200 nm, (**C**) 500 nm, (**D**) 1 µm polystyrene particles.

AFM images of polystyrene particles of different sizes (100, 200, 500 and 1000 nm) were obtained (Figure 4). As shown in the figure, all particles are spherical in shape, and the size corresponds to the data provided by the manufacturer. Some shape deviation and distortion is both due to tip-induced defects and likely partial removal of the particles during scanning.

**Figure 5:**
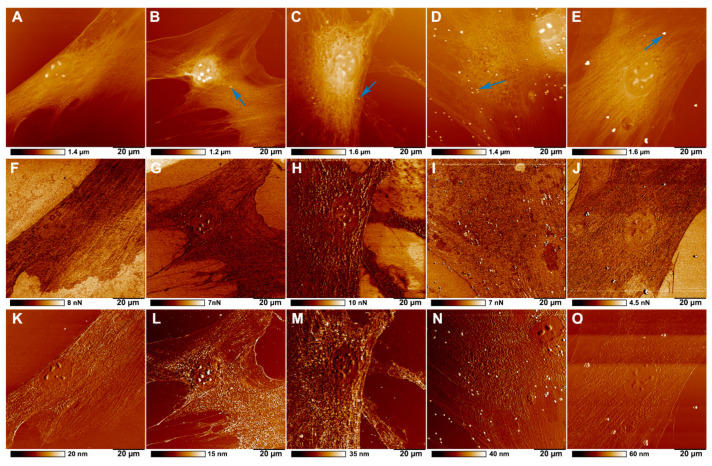
AFM images of topography (**A**–**E**), adhesion (**F**–**J**), and deformation (**K**–**O**) showing the distribution of polystyrene particles in HSF after 24 h co-incubation: control (**A**,**F**,**K**); 100 nm (**B**,**G**,**L**); 200 nm (**C**,**H**,**M**); 500 nm (**D**,**I**,**N**); and 1000 nm (**E**,**J**,**O**).

The penetration of different sized polystyrene particles into the HSF cells was evaluated using AFM. Figure 5 shows the surface topography, and nanomechanical characteristics of HSF incubated with polystyrene nanoparticles. Since AFM visualizes only the sample’s surface, it is challenging to recognize which particles are located outside and inside the cells using the surface topography data. As long as PeakForce Tapping mode is a nanomechanical imaging mode [24], the mechanical features (adhesion and deformation) were obtained for each sample along with topography images by conversion of force curves by applying physical models, such as Hertzian or Derjaguin–Müller–Toporov (DMT) models or extracting data from target points. The 100 nm and 200 nm particles penetrate the cell (which is confirmed in our dark-field hyperspectral microscopy experiments, Figure 3), however, it is difficult to define whether the particles have penetrated the cell utilizing AFM (topography and mechanical properties). We therefore conclude that the practical resolution of AFM is insufficient to detect particles below 200 nm in fixed cells under air imaging conditions. On the contrary, particles with a diameter of 500 and 1000 nm can clearly be resolved in the topography (Figure 5D,E), adhesion (Figure 5I,J) and deformation channels (Figure 5N,O), giving the possibility to identify the uptake of larger particles by cells accurately.

**Figure 6:**
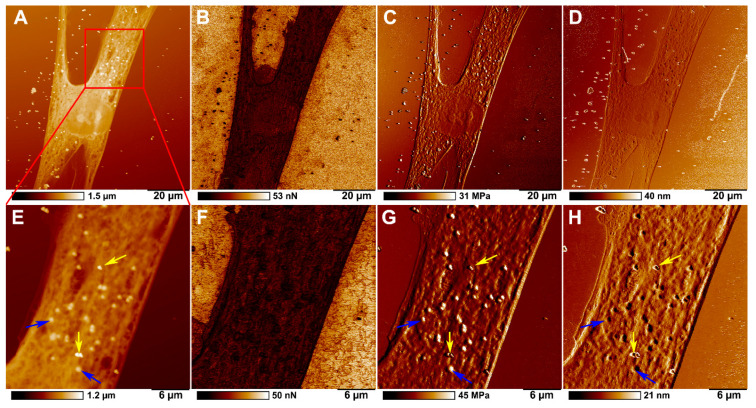
AFM images of topography (**A**,**E**), adhesion (**B**,**F**), Young’s modulus (DMT modulus) (**C**,**G**), and deformation (**D**,**H**) showing the distribution of 500 nm polystyrene particles in HSF after 24 h co-incubation. The yellow arrows indicate polystyrene particles on the cell surface, and the blue arrows indicate particles inside the cells.

**Figure 7:**
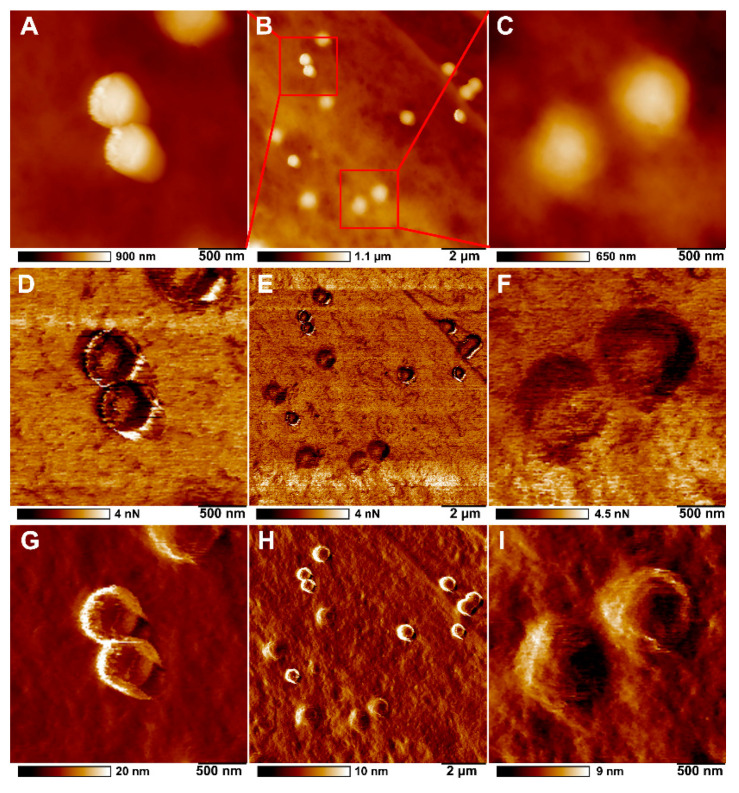
AFM images of topography (**A**–**C**), adhesion (**D**–**F**) and deformation (**G**–**I**) showing the distribution of 500 nm polystyrene particles in HSF after 24 h co-incubation. (**B**,**E**,**H**) The region of cell with polystyrene particles (**A**,**D**,**G**) on the surface and (**C**,**F**,**I**) inside the cell.

Figure 6 shows images of topography, adhesion, Young’s modulus (DMT modulus) and surface deformation of HSF treated with 500 nm polystyrene particles. Nonspecific adhesion was similar in both control and polystyrene-ingested fibroblasts. At the same time, the most notable differences between particles inside and outside the cells were observed in Young’s modulus, obtained by fitting the DMT model, height, and deformation channels. Young’s modulus characterises the material’s stiffness in Pascals, while the deformation channel demonstrated the maximum deformation of the material in nanometres caused by the AFM tip during the approaching mode. It is seen in the height channel that some particles on the cell sample are sharper than others (Figure 6A,E). At the same time, the apparent differences between particles on the cell surface and inside the cells can be seen in the deformation channel (Figure 6D,H). In Figure 6, the yellow arrows indicate particles on the cell membrane surface, while the blue arrows indicate the particles inside the cells. The difference between particles outside and inside the cells could be seen more clearly on magnified images of the cellular surface (Figure 7). It is shown that external particles (Figure 7A,D,G) have sharper boundaries, while the boundaries of internal particles are smoother (Figure 7C,F,I). Although the measured adhesion values for the particles outside and inside cells were significantly different from those of the cell surface itself, but not each other, particles on the surface of the cells had larger deformation values than particles within the cells and the cell itself (Table 1). However, measured DMT modulus values, which characterises the stiffness of individual particles and cell membrane, did not differ significantly, but phase images from this channel still demonstrate similar differences between particles inside cells and particles on the cell membrane (Figure 6C,G). It should also be noted that even though all specimens imaged confirmed the cellular membrane localisation, we were unable to detect intracellular localisation of 1000 nm particles in human skin fibroblasts.

## 3. Discussion

In this Communication we report our first results on the use of PeakForce Tapping nanomechanical AFM to identify submicron polystyrene particles in cultured human cells. An increasing number of in vitro studies aimed at evaluation of microplastics toxicity require an effective yet simple method to detect particles uptake without any use of fluorescent or other labels [23]. AFM has already shown its potential for nanoplastics identification in combination with infrared spectroscopy applied for chemical identification of the unknown environmental particles. Here we utilized a somewhat simpler model, where we used known particles, which were first imaged and then differentiated from other cellular components of similar size and shape using nanomechanical mapping. Using modulus and deformation data, we managed to identify 500 nm particles located within the cytoplasm and on the cellular membrane, similarly to our previous study with clay nanotubes [34]. As confirmed using dark-field hyperspectral microscopy, polystyrene nano- and microbeads are taken up by the cells, aggregation occurs to a certain extent, however most of the beads exists in the cytoplasm as well-isolated individual particles. Microplastics toxicity studies require a suitable approach to differentiate between ingested and surface-attached particles, here we show that surface-attached particles can be detected due to their higher deformation values, whereas the internalized cells exhibit lower values (close to those of cell membrane). Importantly, this differentiation does not require any specific labels or cells sectioning. The direct application of this technology (that is, mapping the biodistribution of polystyrene nano- and microbeads in cell cultures subjected to in vitro microplastics toxicity studies) can be expanded to microplastics of other chemistry. AFM has been used previously to investigate the mechanical properties (including stiffness and adhesion) of polymeric materials. It is obvious that micro- and nanoplastics stiffness and adhesion depend on the chemical composition of the polymer. Hence, the use of standardized stiffness and adhesion data on etalon microplastics will help to identify more than one type of polymers in the specimens. These experiments along with the use of other types of human cells are currently underway in our group and will be reported in the follow-up study.

## 4. Materials and Methods

### 4.1. Fibroblasts Cell Culture

Primary human skin fibroblasts (HSF) were collected from adult skin biopsy with the informed donor consent according to the procedure protocols approved by the Kazan Federal University Local Ethics Committee. The cells were cultured in Eagle’s minimum essential medium (a-MEM; Sigma-Aldrich, Darmstadt, Germany), supplemented with 10% foetal bovine serum (FBS; Sigma-Aldrich), 100 IU/mL penicillin, 100 μg/mL streptomycin, and 2 mM L-glutamine. HSF were incubated under the standard culture conditions in a humidified incubator with 5% CO_2_ at 37 °C, and the growth medium was replaced every 3–4 days [35].

### 4.2. Fibroblasts Viability Evaluation

To observe whether microplastic particles affect cell viability, the 3-[4,5-dimethylthiazol-2-yl]-2,5-diphenyl tetrazolium bromide (MTT) assay was performed accordingly to the manufacturer’s protocol (Sigma-Aldrich, Darmstadt, Germany) [36]. Briefly, fibroblasts were plated onto a 96-well plate at a density of 7 × 10^3^ cells/well. After 24 h of incubation, the cells were exposed to 1, 5 and 10 µg/mL of each microplastic sample for 24 h. The next day, HSF were washed with phosphate-buffered saline (PBS) followed by the addition of 200 μL of fresh medium and 20 μL of MTT reagent. After 4 h of incubation at 37 °C, the culture medium was replaced with 200 μL of dimethyl sulfoxide (DMSO). The optical density values were measured at 540 nm using Multiskan™ FC Microplate Photometer (Thermo Fisher Scientific, Waltham, MA, USA).

### 4.3. Hyperspectral Enhanced Dark-Field Microscopy

Hyperspectral enhanced dark-field microscopy was performed to evaluate the distribution of PS particles in HSF. Upon reaching 80% confluency, the cells at a density of 5 × 10^4^ cells per well were seeded onto 6-well plates containing round glass coverslips. After 24 h of incubation, the cells were exposed to PS particles of each diameter at a 10 µg/mL concentration for 24, 48, or 72 h. After incubation, the cells were washed three times with PBS and then fixed in 4% paraformaldehyde (PFA) for 15 min. The samples were consistently washed with PBS and ultrapure water, and then part of the samples was sealed with Mowiol mounting medium (Sigma-Aldrich), while others were set aside for fluorescence and AFM imaging [37]. The pure samples of PS particles were also prepared to collect endmember spectra. For that, a drop of the aqueous solution of particles (10 µg/mL) was applied directly on a glass slide, and nail polish was used to seal the coverslip. Enhanced dark-field images and hypercubes of PS particles and exposed cells were obtained using the optical system consisting of Olympus BX51 upright microscope (Olympus, Tokyo, Japan) with a motorised stage module ProScan III (JH Technologies, Fremont, CA, USA); a CytoViva^®^ enhanced dark-field condenser (CytoViva, Auburn, AL, USA) with a halogen light source (Fiber-Lite DC-950, 150 W; Dolan Jenner Industries Inc., Boxborough, MA, USA); an ImSpector V10E spectrograph (Specim, Oulu, Finland); and a Pco.Pixelfly usb CCD camera (PCO AG, Kelheim, Germany). Dark-field images and image sequences during fine focus adjustments were obtained using Exponent 7 image acquisition software (Dage-MTI, Michigan City, IN, USA) at 0.1–1.0 s and 5.0% of exposure time and gain value, respectively [38,39]. Then the hypercubes of the same field of view were captured in the range between 400 and 1000 nm with 2 nm spectral resolution at 0.25 s exposure and full illumination intensity. Hyperspectral imaging data were stored and analysed using ENVI v. 4.8. software (Harris Geospatial Solutions, Broomfield, CO, USA) [22,40]. Spectral Angle Mapper (SAM) algorithm that measures the similarity between endmember spectra and spectra found in each pixel of an image was used to verify and map the particles in exposed cells [39]. The similarity is evaluated by calculating the angle between spectrum vectors in space with dimensionality equal to the number of bands. If the angle between the vectors is small enough, the spectra are considered similar to the reference. The spectral profiles (n = 10) of PS particles with a diameter of 100, 200, 500, and 1000 nm were selected in hypercubes using ENVI software’s Region of Interest tool. The mean spectra of selected particles were used to build a spectral library applied as an endmember spectrum in SAM classification. SAM algorithm was used in Single Value mode with Maximum Angle set at 0.4 radians. The classification results were overlaid with the original HSI data using ENVI software, obtaining images with overlaid colour on the classified pixels: red colour for 100 nm; green for 200 nm; blue for 500 nm; yellow for 1000 nm.

### 4.4. Fluorescence Microscopy

Fluorescence images of cells incubated with 500 nm polystyrene particles were obtained using Axio Imager Z2 upright microscope (Zeiss, Göttingen, Germany). F-actin and cell nuclei of fixed cells were stained using DAPI (300 nM; Sigma-Aldrich) and phalloidin-TRITC (50 μg/mL; Sigma-Aldrich) according to the manufacturer’s protocols. The samples were washed consecutively three times with PBS and ultrapure water and then sealed with Mowiol mounting medium (Sigma-Aldrich).

### 4.5. Atomic Force Microscopy

AFM images of polystyrene submicron particles and fixed cells were obtained using a Dimension Icon microscope (Bruker, Billerica, MA, USA) [34,41]. For the AFM imaging, the colloidal suspension of particles was diluted with ultrapure water, and 5 µL of the solution was deposited onto a clean glass slide with subsequent drying at room temperature. Operating was performed in the air in PeakForce Tapping mode using standard silicon nitride cantilevers ScanAsyst-Air (Bruker; nominal length 115 μm, tip radius 2 nm, spring constant 0.4 Nm^−1^) [42]. PeakForce Tapping mode is based on the registration of the interaction force curve at each pixel of the image. The force curve describes the dependence of interaction force between cantilever and specimen on the distance between them. The different mechanical properties of the specimen may be calculated by analysing this curve, including adhesion force or deformation. Since the processing of force curves and their analysis occurs immediately during the scanning, it allows obtaining the information about surface topography and its mechanical parameters simultaneously without further postprocessing [43].

The optimal scanning parameters were set to obtain high-quality images of topography and nanomechanical characteristics: 1–2 nN scanning force at a scanning rate in the 0.8–0.9 Hz range. Images were acquired at a resolution of 512 lines per scan. For each sample were obtained at least 10 AFM scans of cells. The raw AFM data were processed using Nanoscope Analysis v 3.0 software (Bruker) [44]. At least 50 AFM image sections of the surface area of 400 × 400 nm^2^ were used to measure adhesion, Young’s modulus and deformation of individual particles and cell surface.

### 4.6. Statistical Analysis

Statistical analysis was conducted using GraphPad Prism v 8.4.3 (GraphPad Software, Inc., San Diego, CA, USA). Data are expressed as mean values ± SD. The statistical significance for the MTT assay and nanomechanical properties was performed using a parametric one-way ANOVA followed by Dunnett’s or Tukey multiple comparisons post-hoc tests, respectively (level of significance *p* < 0.05).

## Figures and Tables

**Table 1:** Nanomechanical parameters (adhesion, DMT modulus, and deformation) of HSF surface and 500 nm polystyrene outside and inside cells.

	Adhesion (nN)	DMT Modulus (MPa)	Deformation (nm)
Cells	3.2 ± 0.2	120.3 ± 19.5	7 ± 0.2 ***
500 nm PS on cells	2.3 ± 0.1 ***	135.2 ± 19.7	10 ± 0.5 ***
500 nm PS in cells	2.6 ± 0.3 ***	135.7 ± 30.9	8 ± 0.4 ***

*** *p* < 0.001 between groups using one-way ANOVA followed by Tukey’s test. Adhesion: Cells vs. PS on cells, Cells vs. PS in cells (*** *p* < 0.001); Deformation: Cells vs. PS on cells, Cells vs. PS in cells, PS on cells vs. PS in cells (*** *p* < 0.001).

## Data Availability

The data presented in this study are available on request from the corresponding author. The data are not publicly available due to privacy.

## Supplementary Materials

The following are available online at https://www.mdpi.com/article/10.3390/ijms23020806/s1.
