# Supplementary material for: Nanomechanical Atomic Force Microscopy to Probe Cellular Microplastics Uptake and Distribution
Source: Int J Mol Sci. 2022 Jan 12;23(2):806. doi: 10.3390/ijms23020806 (PMC8775627; doi:10.3390/ijms23020806)
Supplement: Supplementary file 1 [file ijms-23-00806-s001.zip › ijms-1486234-supplementary.pdf]

Supplementary Information

Nanomechanical atomic force microscopy to probe cellular microplastics uptake and distribution

Farida Akhatova, Ilnur Ishmukhametov, Gölnur Fakhrullina and Rawil Fakhrullin *

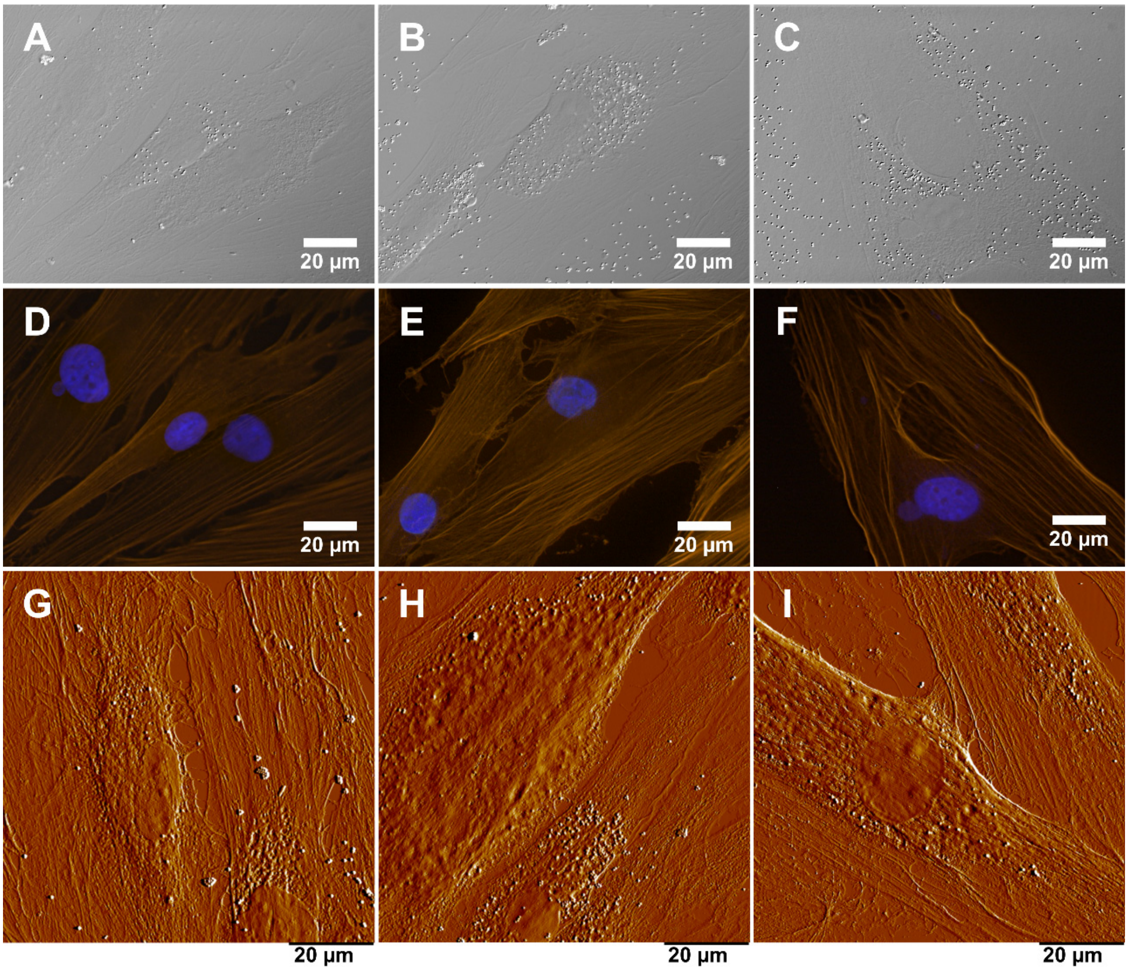

Supplementary Figure S1. Cellular morphology during long-term incubation with 500 nm polystyrene particles. (A-C) Bright-field, (D-F) fluorescence, and (G-I) atomic force microscopy images of human skin fibroblasts after (A-G) 24, (B-H) 48, and (C-I) 72 h of incubation with 500 nm microplastic particles at the 10 µg/mL concentration. F-actin (orange) and nuclei (blue) of cells were stained with phalloidin-TRITC and DAPI, respectively.

Supplementary Video S1. Dark-field image sequence of focus adjusting of human skin fibroblasts incubated with 500 nm polystyrene particles at the 10 µg/mL concentration. Exposure time – 0.2 s.

Supplementary Video S2. Dark-field image sequence of focus adjusting of human skin fibroblasts incubated with 500 nm polystyrene particles at the 10 µg/mL concentration. Exposure time – 1.0 s.
